# Dr. G. K. McGregor

**Published:** 1886-04

**Authors:** 


### MEMORIAL.

### DR. G. K. M'GREGOR, OF WACO, TEXAS.

The subject of this sketch, Dr. G. K. McGregor, recently of Yellow Prairie, deceased, was born in Scotland, of good old Presbyterian parents, on the 12th day of May, 1839, A. D. While quite a child, he came with his parents and settled in North Carolina. In the common schools of the State he received a fair education, as served to establish in him the character of reliability—true to every obligation. At an early age he responded to the calls of his State and country, entered the service of the Confederate States, and, supported by confidence in the justness of the cause, he served as a soldier faithfully and well so long as any effort was demanded. He was actively engaged many times, and on one occasion received a gun shot wound through the right lung, which might have resulted fatally in one less robust and vigorous of constitution. Peace again restored to the country, young McGregor began the study of medicine with Dr. H. McLane, of North Carolina, which he continued, after coming to Texas, with his cousin, Dr. G. C. McGregor.

Constant and faithful to his course, and obliging, he was soon qualified to attend the more enlarged field of instruction afforded by the New Orleans school of Medicine and Surgery. After the required course of study, he was graduated as Doctor of medicine on the 15th of March 1870, A. D. Returning to Texas, he settled near Brenham, became a member of the "Medical Association of Washington county," as also of the "State Medical Association." "Ever faithful to the claims of his profession," as true to every relation of life, he enjoyed the respect and confidence of all who knew him. Too modest to be obtrusive, ambitious only to do good, he was known and loved most by the circle of friends who received his daily attentions. In the spring of 1883 the Doctor began the practice in what is known as "Yellow Prairie, Burleson county, Texas, and on the 25th day of April in same year was married to Miss Carrie Adams, then of Bryan, Texas, whose rare mental culture, and steady Christian walk, secured to him peace and comfort at home with steady trust and hopeful confidence in the promises of God to the faithful worker.

Here, as elsewhere, friends and influence grew upon him, until recognizd as a central and prominent man in his community, illustrating the power and worth of sound principles as the inspiration to life's work. But with all the advantages and prospects here presented, we are called to mourn a life too short to realize the just expectations. The arduous labor incident to a large and growing practice, in an intensely malarious atmosphere, developed inflammation of the liver, obstruction of renal circulation, with suppression of secretion which threatened rapidly fatal termination, (the latter condition was relieved by electricity in a few hours, so as to inspire hope of recovery.) The liver continued to give trouble, however, until abscess discharging into the duodenum, with nausea, vomiting, great prostration, with intolerance of food until death, which came to his relief on the 12th day of October, 1884.

In G. K. McGregor the community lost a true and esteemed citizen, the profession an honorable member, his family a support and trusted councilor, whose place we can scarcely hope to see filled; whose work and good example will long continue to follow, and bless those who are mindful to come after him.

A FELLOW LABORER AND FRIEND.

